# *Plasmodium knowlesi* (*Pk*) Malaria: A Review & Proposal of Therapeutically Rational Exchange (T-REX) of *Pk*-Resistant Red Blood Cells

**DOI:** 10.3390/tropicalmed8100478

**Published:** 2023-10-20

**Authors:** Ryan Philip Jajosky, Shang-Chuen Wu, Philip G. Jajosky, Sean R. Stowell

**Affiliations:** 1 Joint Program in Transfusion Medicine, Brigham and Women’s Hospital, Harvard Medical School, 630E New Research Building, 77 Avenue Louis Pasteur, Boston, MA 02115, USA; swu26@bwh.harvard.edu (S.-C.W.);; 2 Biconcavity Inc., Lilburn, GA 30047, USA

**Keywords:** global health, tropical disease, infectious disease, protozoa, *Plasmodium knowlesi*

## Abstract

*Plasmodium knowlesi* (*Pk*) causes zoonotic malaria and is known as the “fifth human malaria parasite”. *Pk* malaria is an emerging threat because infections are increasing and can be fatal. While most infections are in Southeast Asia (SEA), especially Malaysia, travelers frequently visit this region and can present with *Pk* malaria around the world. So, clinicians need to know (1) patients who present with fever after recent travel to SEA might be infected with *Pk* and (2) *Pk* is often misdiagnosed as *P. malariae* (which typically causes less severe malaria). Here we review the history, pathophysiology, clinical features, diagnosis, and treatment of *Pk* malaria. Severe disease is most common in adults. Signs and symptoms can include fever, abdominal pain, jaundice, acute kidney injury, acute respiratory distress syndrome, hyponatremia, hyperparasitemia, and thrombocytopenia. Dengue is one of the diseases to be considered in the differential. Regarding pathophysiologic mechanisms, when *Pk* parasites invade mature red blood cells (RBCs, i.e., normocytes) and reticulocytes, changes in the red blood cell (RBC) surface can result in life-threatening cytoadherence, sequestration, and reduced RBC deformability. Since molecular mechanisms involving the erythrocytic stage are responsible for onset of severe disease and lethal outcomes, it is biologically plausible that manual exchange transfusion (ET) or automated RBC exchange (RBCX) could be highly beneficial by replacing “sticky” parasitized RBCs with uninfected, deformable, healthy donor RBCs. Here we suggest use of special *Pk*-resistant donor RBCs to optimize adjunctive manual ET/RBCX for malaria. “Therapeutically-rational exchange transfusion” (T-REX) is proposed in which *Pk*-resistant RBCs are transfused (instead of disease-promoting RBCs). Because expression of the Duffy antigen on the surface of human RBCs is essential for parasite invasion, T-REX of Duffy-negative RBCs—also known as Fy(a-b-) RBCs—could replace the majority of the patient’s circulating normocytes with *Pk* invasion-resistant RBCs (in a single procedure lasting about 2 h). When sequestered or non-sequestered iRBCs rupture—in a 24 h *Pk* asexual life cycle—the released merozoites cannot invade Fy(a-b-) RBCs. When Fy(a-b-) RBC units are scarce (e.g., in Malaysia), clinicians can consider the risks and benefits of transfusing plausibly *Pk*-resistant RBCs, such as glucose-6-phosphate dehydrogenase deficient (G6PDd) RBCs and Southeast Asian ovalocytes (SAO). Patients typically require a very short recovery time (<1 h) after the procedure. Fy(a-b-) RBCs should have a normal lifespan, while SAO and G6PDd RBCs may have mildly reduced half-lives. Because SAO and G6PDd RBCs come from screened blood donors who are healthy and not anemic, these RBCs have a low-risk for hemolysis and do not need to be removed after the patient recovers from malaria. T-REX could be especially useful if (1) antimalarial medications are not readily available, (2) patients are likely to progress to severe disease, or (3) drug-resistant strains emerge. In conclusion, T-REX is a proposed optimization of manual ET/RBCX that has not yet been utilized but can be considered by physicians to treat *Pk* malaria patients.

## 1. Background

In 1927, *Plasmodium knowlesi* (*Pk*) was first observed by Giuseppe Franchini when examining the blood of *Macaca fascicularis* (long-tailed macaque, or cynomolgus monkey) [1]. In 1931, Napier and Campbell inoculated *Pk* into *M. mulatta* (rhesus macaques) and described severe infection. In 1932, B. M. Das Gupta and Robert Knowles described experimental *Pk* infection in humans [2]. Consequently, *Pk* was named after Knowles. *Pk* is sometimes referred to as the “fifth human malaria parasite”, after *P. falciparum* (*Pf*), *P. vivax* (*Pv*), *P. ovale*, and *P. malariae* (*Pm*) [3,4,5,6]. *P. ovale* is now divided into *P. ovale wallikeri* and *P. ovale curtisi*. This is an example of sympatric speciation, in which both parasites co-exist in the same location, but the reason for this divergence is not fully understood [7]. 

**Figure 1:**
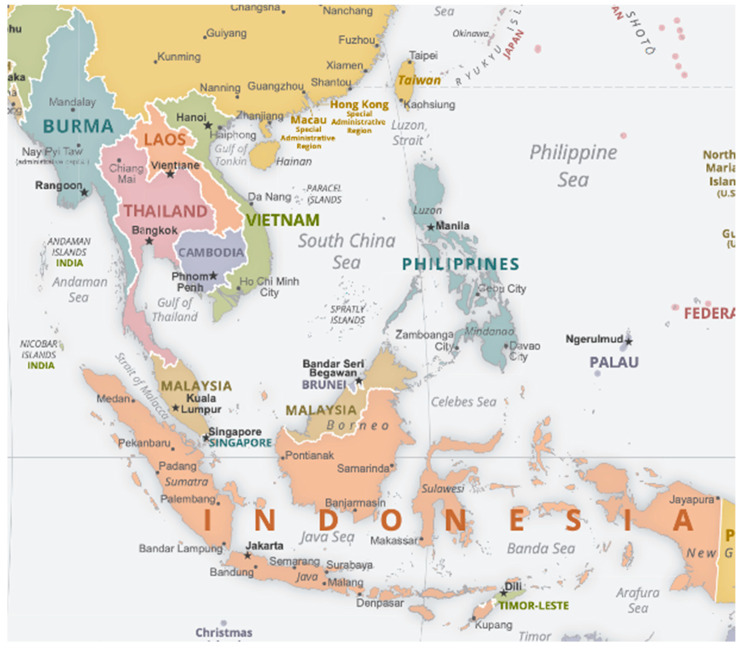
Political map of SEA obtained from The World Factbook [31].

**Figure 2:**
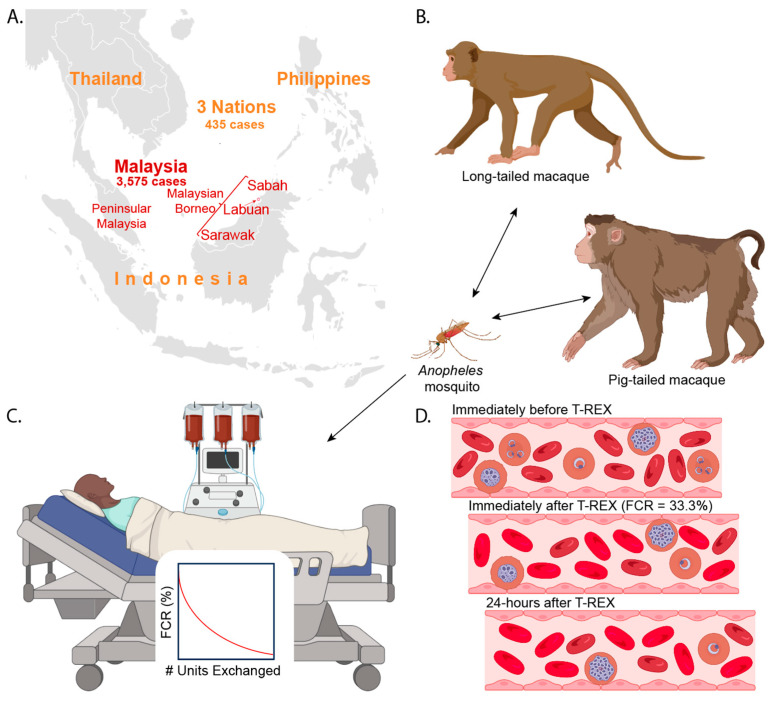
(**A**) Nations with *Pk* malaria cases described in the 2022 World Health Organization (WHO) Malaria Report [32]. In 2021, there were 3575 *Pk* cases in Malaysia and 435 total cases in Thailand, the Philippines, and Indonesia. The map of SEA is in the public domain and was obtained from Wikimedia Commons and then edited [33]. (**B**) *Anopheles* mosquitoes can transmit *Pk* from macaques to macaques or humans. (**C**) A *Pk* malaria patient being treated with adjunctive RBCX, which reduces the number of pre-RBCX RBCs. The extent of the reduction is determined by the procedure settings and is technically known as the fraction of (original) cells remaining (FCR). For malaria patients, the FCR reflects the fraction of the pre-RBCX circulating iRBCs remaining since RBCX cannot remove the sequestered iRBCs. (**D**) Immediately before T-REX, the patient has circulating uRBCs, iRBCs, and sequestered iRBCs. Immediately after T-REX with an FCR of 33.3%, 66.6% of the patient’s original circulating uRBCs and iRBCs are removed, but the sequestered iRBCs are not. In this schematic, *Pk* invasion-resistant Fy(a-b-) RBCs were transfused during T-REX (depicted as brighter colored RBCs). After one *Pk* asexual life cycle, which is 24 h, the sequestered and non-sequestered iRBCs will rupture, releasing free merozoites that cannot invade Fy(a-b-) donor RBCs. Multiple images were obtained from BioRender.

*Pk* primarily infects individuals in Southeast Asia (SEA) (Figure 1) [8,9]. Malaysia has the most cases of any nation (Figure 2A), and Peninsular Malaysia has fewer cases than Malaysian Borneo (containing the states of Sarawak, Sabah, and the Federal Territory of Labuan) [9]. In 2020, Malaysia had 2839 malaria cases, of which 1537 (54.1%) were in Sabah and 862 (30.4%) were in Sarawak, and the vast majority were caused by indigenous *Pk* [10]. In addition, travelers to SEA can acquire *Pk* infection and then require medical care around the world [11,12,13,14].

Of the five major *Plasmodium* species that cause malaria in humans, *Pk* is the only one with zoonotic transmission and is known as a simian malaria parasite [15,16]. The predominant natural hosts of *Pk* are *M. fascicularis* and *M. nemestrina* (pig-tailed macaque) (Figure 2B) [17,18]. Macaque–mosquito–macaque transmission and macaque–mosquito–human transmission occur naturally by *Anopheles* of the Leucosphyrus Group, which are exophagic (bite outdoors) [19,20,21,22]. These mosquitoes are found in forests and forest edges, where adult men often work as farmers, loggers, hunters, etc., which helps explain higher infection rates in males [23,24]. (Note: *An. gambiae* is the chief mosquito vector of *Pf* [25].) The dominant vector of *Pk* in Sabah is *An*. *balabacensis,* while *An. latens* is the main vector in parts of Sarawak [18,26]. Experimental human–mosquito–human transmission has been demonstrated by *An. balabacensis* [27], but sustained natural transmission is thought to be unlikely [28]. 

Timor Leste is the only nation in SEA to not report a case of human *Pk* malaria [8]. While the explanation is unclear, articles from 2013 and 2016 show that Timor Leste is not home to *Anopheles* in the Leucosphyrus Group [22,29]. Instead, Timor Leste is home to *An. barbirostris* and *An. subpictus* [30]. *Pf* was the dominant species in Timor Leste. In June 2017, the last indigenous case of malaria was reported in Timor Leste. 

Interestingly, early in the 20th century, both *Pk*-infected RBCs (*Pk* iRBCs) and *Pv* iRBCs were used as a pyretic agent to treat neurosyphilis [34]. This strategy was supported by in vitro data showing that *Treponema pallidum* could be killed at temperatures that are reached during human fevers [34], subsequent data that *T. pallidum* does not have a heat-shock response [35], and data that it has a heat-labile 3-phosphoglycerate mutase [36]. Julius Wagner-Jauregg, an Austrian psychiatrist, received the 1927 Nobel Prize in Physiology or Medicine for developing this treatment, although its efficacy has been questioned in modern times because there were no randomized controlled trials [34]. *Pk*-iRBC malariotherapy was abandoned when the risks were recognized [4,8,29,37,38]. Currently, the Centers for Disease Control and Prevention (CDC) in the United States of America (US) has warned about the severity of *Pk* malaria and the need to immediately start appropriate treatment [39]. In terms of whether fever is beneficial for *Pf* infection, it was recently discovered that *Pf* transcription factor PfAP2-HS leads to transcription of *hsp70-1* and *hsp90*, which protects it from high temperatures [40].

## 2. Pathophysiology

Although both *Pk* and *Pf* are associated with an elevated risk of severe malaria [41], the pathophysiology has notable similarities and differences.

### 2.1. Pk Life Cycle

The *Pk* life cycle resembles that of other human-infecting *Plasmodium*, i.e., involving a mosquito, liver, and blood stage [29]. When *Plasmodium* infected *Anopheles* mosquitoes take a blood meal from a person, *Plasmodium* sporozoites are injected into the bloodstream. The sporozoites invade hepatocytes, develop into schizonts, and then rupture and release merozoites, which invade normocytes and/or reticulocytes. Asexual multiplication (erythrocytic schizogony) occurs when immature trophozoites (ring forms) develop into mature trophozoites and then into schizonts that rupture and release merozoites [42]. A small fraction of intraerythrocytic parasites develop into their sexual forms (gametocytes), through a process known as gametocytogenesis [43]. Sexually committed ring forms can be indistinguishable from asexual ones [43]. The male and female forms are known as microgametocytes and macrogametocytes, respectively. The malaria life cycle is described in detail elsewhere [29,42]. Importantly, the clinical signs and symptoms are caused by the erythrocytic stage. Notably, *Pk* does not have a latent (dormant) liver stage involving hypnozoites.

### 2.2. RBC Invasion

*Pk* and *Pf* invasion of RBCs occurs in about 60 seconds [44]. After a merozoite binds to a human cell, it orients so the host cell is facing the parasites’ apical end, containing micronemes and rhoptries, which are secretory organelles. The parasite’s apical end then fuses with the RBC, and the actomyosin motor advances the parasite into the RBC. This invaginated RBC membrane is known as the parasitophorous vacuole. Duffy binding proteins (DBPs) and reticulocyte binding-like proteins (RBLs) are used during invasion [44]. 

Both *Pk* and *Pf* merozoites use Apical Membrane Antigen 1 (AMA1 or AMA-1) to reorient the parasite’s apical end toward the host cell [45,46]. Anti-*Pk*AMA1 IgG antibodies can block *Pk* invasion into human RBCs in vitro [47]. Because AMA-1 is highly immunogenic and has low levels of polymorphisms, it has been a target for *Pk* and *Pf* vaccine development [48,49,50,51]. *Pk* merozoites require the Duffy antigen (also known as Atypical Chemokine Receptor 1 (ACKR1) or CD234) [52] on human cells for invasion, which is similar to *Pv*, while *Pf* requires basigin (CD147) and CD55 [5,53,54,55]. 

For *Pk* to invade human cells, it requires normocyte-binding protein Xa (*Pk*NBPXa) and the second region of Duffy-binding protein α (*Pk*DBPαII) [5,56]. Both proteins are targets for vaccine development [5,57]. The human receptor for *Pk*NBPXa is unclear. In contrast, *Pk*NBPXa and b as well as *Pk*DBPα, β, and γ can bind to macaque cells [57]. *Pk*NBPXa is required for invasion of human cells, but not macaque cells, possibly because *Pk*NBPXb can compensate [44]. *Pk*DBPβ and γ only bind to macaque cells because they display *N*-glycolylneuraminic acid (Neu5Gc), while human cells do not [58,59]. Although *Pv* also uses a DBP (*Pv*DBPII) to invade human cells, it has a much stronger preference for reticulocytes than normocytes [57]. One reason is that it expresses *Pv*RBP2b, which binds to the reticulocyte marker CD71 (transferrin receptor 1). *Pf* requires *Pf*Rh5 to invade human cells expressing basigin [44].

*Pk* and *Pf* parasites can invade both reticulocytes and normocytes [57,60,61,62]. One study evaluated reticulocyte and normocyte invasion by *Pk* strain A1-H.1. The H strain was obtained from the first person with confirmed natural infection. From the H strain, the A1 strain was derived and maintained in vivo in rhesus macaques. Then, it was cultured in vitro in macaque and human RBCs until it adapted to survive exclusively in human RBCs [63]. This is how the A1-H.1 strain was created. This strain invaded 2.2% of human normocytes and 8.6% of reticulocytes in an in vitro assay [60]. The *Pk* strain UM01 was also tested. It was derived from a patient treated in Malaysia and expanded in vivo in *M. fascicularis*. This strain invaded 2.1% of human normocytes and 2.9% of reticulocytes [60]. Because a normal reticulocyte count is 0.5–2.5% in healthy adults, mature RBCs are far more abundant in the peripheral blood and likely contribute to the disease pathogenesis [64]. 

### 2.3. Pk Intraerythrocytic Cycle

Although *Pk*’s intraerythrocytic replication cycle takes approximately 24 h, compared to 48 h for *Pf* and *Pv*, median parasitemia was lower in hospitalized *Pk* patients than in *Pf* or *Pv* patients [29,63,65,66,67]. One group studied the baseline characteristics of non-pregnant malaria patients ≥ 15 years old who presented to a hospital in Malaysia [66]. They found the median parasite density was 1387 (*n* = 107), 26,781 (*n* = 24), and 4258 (*n* = 21) parasites per microliter for *Pk*, *Pf*, and *Pv* patients at presentation, respectively. This seems surprising given that *Pk* has the fastest asexual life cycle. Despite *Pk* patients having a lower parasite density, they had similar clinical and laboratory findings to the *Pf* and *Pv* patients [66]. They also found the mean age was 44.9, 38.7, and 35.5 years for *Pk*, *Pf*, and *Pv* patients, respectively. In addition, the mean platelet count was 71,000, 108,000, and 118,000 platelets per microliter for *Pk*, *Pf*, and *Pv* patients, respectively. A different study evaluated baseline characteristics of non-pregnant malaria patients > 12 years old who presented to three different hospitals in Malaysia. Similarly, *Pk* patients were the oldest, had the lowest parasite densities, and had the lowest platelet counts at baseline [65]. In addition, this study found parasite densities > 20,000 parasites per microliter in 15%, 32%, and 7% of *Pk*, *Pf*, and *Pv* patients, respectively. Perhaps *Pk* patients have lower parasite counts than *Pf* patients because they present at hospitals earlier than *Pf* patients—plausible since *Pk* patients, in general, are older and have lower platelet counts than *Pf* patients. 

### 2.4. RBC Deformability

Both *Pk* and *Pf* malaria are associated with reduced RBC deformability of both iRBCs and uninfected RBCs (uRBCs) [68,69]. One study attributed the reduced deformability of uRBCs to the fact that a high proportion of the uRBCs were echinocytes, which have increased membrane shear modulus (resistance to membrane extension) [68]. The median percentage of uRBCs which were echinocytes, was 96% in *Pk* patients and 1% in controls (*p* = 0.0002). This finding was not observed in uRBCs from *Pk*-infected *M. fascicularis*. While the methods describe the use of a Giemsa stain, the photograph of the echinocytes appears to be from an unstained sample on a 37 °C stage in isotonic saline. Thus, this photograph does not resemble a typical thin smear of peripheral blood. Thin smears photographs from *Pk* patients published by the CDC and other sources do not seem to show a dramatic number of uRBCs having echinocyte morphology, and these sources do not mention high percentages of echinocytes [29,70,71]. The authors showed that echinocyte formation was not caused by the cryopreservation and thawing process because a high percentage of echinocytes was also seen with fresh blood samples. The authors did not find a positive correlation between the percentage of echinocytes and the length of time from sample collection to analysis. 

*Pf* researchers have studied the mechanism by which uRBCs become less deformable [72]. One research study proposed that iRBCs release ATP, which is converted to AMP via CD39; AMP is converted to adenosine via CD73; and adenosine signals through the adenosine A2B receptor (ADORA2B) found on RBCs increase intracellular cAMP [72]. The cAMP causes protein kinase A to phosphorylate cytoskeletal proteins, which reduces the RBCs’ deformability. Thus, the extracellular adenosine can act as a “bystander molecule”, reducing RBC plasma membrane deformability *in cis* for iRBCs and *in trans* for uRBCs [72]. Regardless of the mechanism, reduced deformability may contribute to the microvascular accumulation of *Pk*- and *Pf*-iRBCs [68,73,74]. 

### 2.5. Cytoadhesion & SICA Antigens

Although all blood-stage forms of *Pk* circulate, recent data suggest that a subset of *Pk*-iRBCs demonstrate endothelial cytoadherence (sequestration), which is a key mechanism for the microvascular accumulation of *Pf*-iRBCs [74,75,76,77,78]. A recent study of *Pk* malaria in rhesus macaques, a non-natural host, was conducted by the Emory National Primate Research Center [74]. The study found that cytoadherence and sequestration can occur in a subpopulation of *Pk* iRBCs. The sequestration of iRBCs can protect against splenic clearance [79]. However, the sequestration ends when the schizont ruptures to release merozoites [80]. This occurs once every 24 h (the duration of *Pk*’s asexual lifecycle). *Pk* sequestration is likely dependent on Schizont-Infected Cell Agglutination (SICA) antigens. These protein antigens, encoded by the *SICAvar* gene family, are only expressed on iRBCs when a functional spleen is present [74,77]. 

SICA[+] iRBCs, which are highly virulent in rhesus macaques, can be found in rhesus macaques with functional spleens. When SICA[+] iRBCs are passaged in splenectomized rhesus macaques, they become less virulent SICA[-] iRBCs. If SICA[-] iRBCs are passaged in rhesus macaques with function spleens, either a milder disease is observed or SICA[+] iRBCs form and lead to more severe disease. The exact mechanism by which the spleen regulates *SICAvar* gene expression is unknown, and additional research on this topic is needed. *Pf* has analogous variant antigens known as *Pf* erythrocyte membrane protein 1 (*Pf*EMP1), which is encoded by the *var* gene family. *Pf*-iRBCs cytoadhere to endothelial receptors (e.g., CD36, ICAM-1, etc.) via knob-like protrusions, which display *Pf*EMP1 [73]. In contrast, *Pk*-iRBCs do not have knobs which protrude outward, but have caveolae pits (which invaginate) [73]. An autopsy of a person with *Pk* malaria, but not with cerebral malaria (CM), demonstrated neither cytoadherence nor increased intercellular adhesion molecule 1 (ICAM-1) on brain endothelium [78]. In contrast, cytoadherence and the upregulation of ICAM-1 on brain endothelium are evident in cases of *Pf* CM [81]. These findings may explain why *Pf* is associated with CM, while *Pk* is not associated with CM.

### 2.6. Pediatric Patients Rarely Develop Severe Pk Malaria

While *Pk* can infect both children and adults, life-threatening malaria typically spares children [41,65,82,83]. There is a positive correlation between age and *Pk* parasitemia [84]. In *Pk* patients, there is also a direct correlation between age and angiopoietin-2 (a marker of endothelial activation), interleukin-6 (IL-6) (a pro-inflammatory cytokine), and microvascular dysfunction, independent of parasitemia [84]. Both angiopoietin-2 and IL-6 increase with age and increase with *Pf* severity, which may help explain why *Pk* disease severity positively correlates with age [84]. 

### 2.7. Tissue Expression of Duffy Antigen

In *Pk* schizonts, *Pk*DBPα is found within micronemes, a parasite secretory organelle [44]. After *Pk* merozoites egress from human cells, *Pk*DBPα is gradually and continuously secreted onto the merozoite surface [44]. *Pk*DBPα is most abundant at the merozoites’ widest circumference and the tip of the basal end [44]. Importantly, *Pk*DBPα release onto the merozoite surface has been observed even if merozoites do not bind to RBCs [44].

Thus, it is worth considering the localization of Duffy antigen expression in non-erythroid human cells. A 1997 study conducted by researchers at the New York Blood Center examined human tissues using a Duffy-specific antibody. They found Duffy expression on the endothelium in the kidney, lung, spleen, and thyroid [85,86]. In addition, Duffy was expressed on epithelium of the kidney’s collecting ducts and lung alveoli. It has also been found on the Purkinje cells of the cerebellum [86]. This is intriguing because the kidney and lung can be severely damaged in human *Pk* malaria. In addition, studies of *Pk* infection in rhesus macaques found that *Pk* iRBCs were mostly found in the kidney, lung, spleen, gastrointestinal tract, and adrenal gland [74]. It is unclear whether *Pk*DBPα can bind to Duffy antigen in these locations (e.g., renal and pulmonary endothelium). It is also worth noting that *Pv*, which also has a DBP, can be associated with AKI and ARDS [87]. 

## 3. Clinical Course

### 3.1. Signs and Symptoms

*Pk*-infected children less than 15 years old are much more likely to be asymptomatic than adults, and pediatric patients rarely experience severe disease or death [41,65]. Adult *Pk* malaria patients often present with the following non-specific symptoms: fever, chills, headache, abdominal pain, myalgia, nausea, vomiting, and cough [73]. Clinical signs often include tachycardia and tachypnea, while hypotension, jaundice, and respiratory distress may also manifest. Neurologic findings are rare, however retinal hemorrhages can occasionally be seen, likely due to the near universal finding of thrombocytopenia in adults [73]. According to WHO criteria, slightly more than one-third of adults present with anemia. Leukocyte counts are usually normal, except for neutrophilia, which is seen in severe cases. 

Several studies have described signs and symptoms of severe *Pk* malaria. A study of *Pk*-infected patients in Malaysia included 28 cases of severe malaria, which were only seen in adults. The WHO malaria severity criteria that were most often identified in severe *Pk* malaria are as follows: severe acute kidney injury (AKI), jaundice, hyperparasitemia (parasite density > 100,000 parasites/μL), and severe anemia [65]. The study also found that a parasite density > 15,000/μL was the best predictor of severe *Pk* malaria (adjusted odds ratio 16.1, *p* < 0.001). A different report of severe *Pk* malaria in Malaysia included 44 patients [88]. The study found that the WHO malaria severity criteria most often seen in severe *Pk* malaria were as follows: AKI, jaundice, hyperparasitemia, and acute respiratory distress syndrome (ARDS). Coma, which is associated with CM, did not occur in either study. Multiple studies have shown parasitemia has a positive correlation with age and is associated with severe disease [73,84]. 

### 3.2. Deaths

In 2019, a case-series and systematic review of 32 polymerase chain reaction (PCR)-confirmed *Pk* malaria deaths in Malaysia was published [41]. At presentation, the median age was 56, and 56% were men. All fatal cases were in adults, and all presented with thrombocytopenia (median of 38,500 platelets per microliter), increased creatinine, and hyponatremia (median of 128 mmol/L) if lab testing was performed. Thrombocytopenia is also seen in *Pv* and *Pf* malaria patients, but the reason is not fully understood [89]. However, platelets are known to kill malaria parasites [89]. While the underlying cause of hyponatremia is unknown, it might be related to AKI. Hyponatremia can also be seen in severe *Pf* malaria [90]. 

After retrospectively reviewing the patient’s medical records, ~94% of these patients met the WHO criteria for severe malaria, but only 63% of these patients were properly diagnosed with severe malaria at the time of admission. The most common severe malaria criteria that were identified at presentation included jaundice, severe AKI, and respiratory distress. In addition, abdominal pain was reported by 65% of patients, and cardiovascular-metabolic comorbidities were present in 34% of cases. None of the patients that died had severe anemia at presentation. Only 1 of 30 patients who died had coma at presentation; however, alternative causes in this case were not investigated. Interestingly, an autopsy of a patient without coma revealed cerebral pathology, including sequestration in the brain and petechial hemorrhages [78]. 

Why did these patients die? In 90% of the fatal cases, the *Plasmodium* species was misidentified by microscopy, with the vast majority being misdiagnosed as *Pm* (which typically causes milder malaria). Consequently, there was a delay in starting intravenous (IV) artesunate in 36% of cases. When severe malaria had been properly diagnosed at presentation, IV antimalarial medication was provided immediately in 81% of cases. In two cases, IV artesunate was unavailable. The median time to death was 41 h. This study also found that independent risk factors for death included female sex (reason unknown) and age ≥ 45 years, which is a previously described risk factor for severe *Pk* malaria.

## 4. Differential Diagnosis

Based on the signs and symptoms of *Pk* malaria, the differential diagnosis may include arboviruses (dengue virus) [78], influenza [91], pharyngitis [92], Typhoid fever [93], leptospirosis [93], viral encephalitis [11], dyspepsia [41], gastritis [94], rhabdomyolysis [95], and others. Dengue, Typhoid fever, and leptospirosis warrant discussion. 

Dengue viral infections can mimic *Pk* malaria [96]. This is because both are transmitted by mosquitoes in SEA and both can be associated with fever, nausea, vomiting, abdominal pain, muscle aches, headache, hypotension, thrombocytopenia, and hyponatremia [78,97]. Atypical features of dengue can include ARDS, renal failure, rhabdomyolysis, and others [11]. Thus, dengue diagnostic testing (e.g., serology, antigen detection, molecular testing, etc.) is often mentioned in *Pk* malaria case reports [78,98,99,100]. There may be clues to differentiating between the two. For example, the *Aedes aegypti* mosquito, which can transmit dengue virus, is an “urban mosquito”, so most infections are in urban/semi-urban areas [97,101]. Dengue can be associated with prolonged fever, pain behind the eyes, bleeding, leucopenia and increased vascular permeability, which can lead to ascites, pleural effusion, and a progressively increasing hematocrit [102,103]. However, thrombocytopenia can lead to bleeding, which can reduce the hematocrit. Of course, co-infection with *Pk* can also occur [97,104].

Typhoid fever is caused by the bacteria *Salmonella enterica* serotype Typhi and is often confused with malaria [105,106]. Regarding Asia, most cases occur in South Asia (e.g., India, Pakistan, Bangladesh). It is most often spread through sewage that contaminates water or food and can be transmitted from human-to-human. Key signs and symptoms include persistent fever, abdominal pain, nausea, vomiting, headache, diarrhea, constipation, maculopapular rash resembling rose-colored spots on the trunk, hepatosplenomegaly, and others [106,107]. Complications include intestinal hemorrhage, anemia, intestinal perforation, cholecystitis, hepatitis, pneumonia, myocarditis, shock, encephalopathy, and others [107]. Blood culture is the preferred method of diagnosing acute infection, but bone marrow culture can increase the sensitivity [106].

Leptospirosis is caused by *Leptospira* species, which can be acquired in SEA [108], and is often mistaken for malaria [109]. It can be transmitted through the mucous membranes, cuts in the skin, urine, reproductive fluids, or contaminated food or water. Animals such as rats often shed the bacteria in their urine [109]. Outbreaks are associated with heavy rain, flooding, and hurricanes, which spreads the infected urine [109]. For example, travelers to SEA who engage in recreational boating or swimming in contaminated fresh water or are exposed to contaminated mud can become infected. While most infections are asymptomatic, some progress with signs and symptoms including fever, chills, lower-back and calve pain, headache, photophobia, conjunctival suffusion, retro-orbital pain, nausea, vomiting, and skin rash [108]. Severe disease includes cardiac arrhythmia, hemorrhage, jaundice, shock, liver failure, aseptic meningitis, renal failure, pulmonary insufficiency, and pulmonary hemorrhagic syndrome [108]. The diagnosis can be made using PCR of whole blood or cerebrospinal fluid (CSF). CDC states that the microscopic agglutination test (MAT) is the “reference standard” [108]. If serology is used, CDC recommends an IgM-specific screening test, followed by confirmation using the MAT [108].

## 5. Pk Diagnosis

### 5.1. Microscopy

The diagnostic criteria for *Pk* malaria can vary based on location. According to the 2013 Management Guidelines of Malaria in Malaysia, the diagnosis is made using clinical suspicion and the detection of blood parasites [110,111]. The CDC “Algorithm for Diagnosis and Treatment of Malaria in the United States” starts with determining (1) if a patient has a fever and has travelled to a malaria-endemic region or (2) if there is a clinical suspicion of malaria [112]. If either is true, thick and thin blood smears need to be immediately examined using a microscope. The thick smear is used to detect *Plasmodium* parasites, while the thin smear is to identify the species and measure parasitemia [39]. In Malaysia, light microscopy is the first-line method to diagnose *Pk* malaria [113]. The CDC provides blood smear photographs and a table of microscopic features of the iRBCs and parasites for the five major human-infecting *Plasmodium* species [70]. If blood smears do not show *Plasmodium*, they must be repeated every 12–24 h until three sets of smears have been reviewed. 

**Figure 3:**
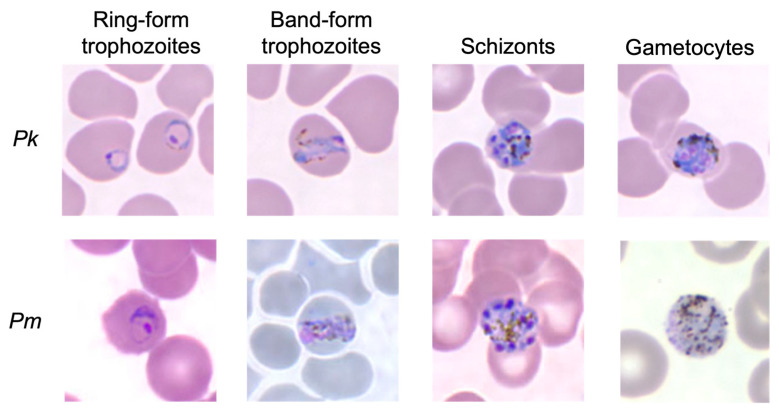
*Pk* and *Pm* can have a similar morphologic appearance. The images were courtesy of DPDx from the Centers for Disease Control and Prevention (CDC) [70].

Early trophozoite forms of *Pk* can resemble *Pf*, while *Pk*’s late/mature trophozoite forms, schizonts, and gametocytes can resemble *Pm* [29,114,115,116]. In addition, a case-series in Thailand described six *Pk* malaria cases, of which five were initially diagnosed with *Pv* [91]. *Pk* is most often mistaken for *Pm* (Figure 3), which typically causes less severe malaria. So, parasites resembling *Pm* are supposed to be reported as *Pk* in areas where *Pk* is prevalent (Sabah and Sarawak) [110,111]. In Malaysia, the definitive diagnosis is made using PCR [117]. A study on the morphology of *Pk* on blood smears found subtle features that may distinguish it from *Pm* [71]. For example, *Pk* trophozoites can have double chromatin dots, 2–3 parasites per RBC, and mature schizonts can have as many as 16 merozoites (versus up to 12 for *Pm*, up to 16 for *P. ovale*, and up to 24 for *Pv* and *Pf*) [70,71]. The authors conclude that it is difficult to identify *Pk* based on morphology. They suggest that a diagnosis of *Pk* malaria can be made based on the following: morphology resembling *Pm*, severe disease, parasite density > 5000/uL, and recent time in forest fringes in SEA [71]. Since 2018, Malaysia has not reported indigenous cases of *Pf*, *Pv*, *Pm*, *nor P. ovale* malaria, but they still have imported cases, which must be distinguished from *Pk* [118]. 

Some researchers have used immunohistochemistry (IHC) to try to detect *Pk* in tissue sections [78]. For example, an autopsy was performed on a patient with PCR-confirmed *Pk* malaria, and brain sections were stained using IHC. The anti-*Pf* histidine-rich protein (HRP) stain was negative, while the anti-*Pf*/*Pv* aldolase stain was positive [119]. Of course, the anti-*Pf*HRP may not cross-react with *Pk* antigens, and the anti-*Plasmodium* aldolase stain is not specific for *Pk*.

### 5.2. Molecular Testing

Fortunately, PCR and molecular testing, the gold-standard for diagnosis, are becoming more accessible in Malaysia and other countries [23,120]. Rapid *Pk* testing can also be performed using loop-mediated isothermal amplification (LAMP), a point-of-care test that is related to PCR, but amplifies nucleic acids at a constant temperature [8,23]. While molecular methods can be very accurate, their limitations include cost and resources (i.e., equipment, reagents, trained operators to perform the tests, etc.).

### 5.3. RDTs

The WHO recommends either microscopy or rapid diagnostic tests (RDTs) to diagnose malaria [121]. RDTs are lateral flow immunochromatographic tests that can identify malaria antigens or antibodies [122,123]. Because there is a lack of microscopy in many parts of SEA, RDTs could fill this void [113]. However, no *Pk* RDT has been developed [113]. In one study, the best performing antibody for detecting *Plasmodium* in *Pk*-infected persons was the anti-*Pv*-parasite lactate dehydrogenase (pLDH) component of the Biocredit^TM^ RDT, with 92% sensitivity [113]. Yet, none of the available RDTs met the 95% sensitivity cutoff that WHO uses to replace microscopy as the first-line method for diagnosis [113]. RDTs which detect *Pf* HRP2 did not cross-react with *Pk* strain A1-H.1, so this RDT could be useful to exclude *Pf*, which can morphologically resemble *Pk* [14,113]. 

There is a need for a *Pk* RDT, especially in locations where molecular diagnostics are not feasible. One potential candidate target may be *Pk* Serine Repeat Antigen 2 (*Pk*SERA3 Ag2), a protein with an unknown function that is expressed in late trophozoites and schizonts [124]. It has been employed for the serological detection of *Pk* infection [125]. The peptide AELQKAKMV in *Pk*SERA3 Ag2 has recently been shown to be specific for *Pk* and not for other human-infecting *Plasmodium* species [124]. Of note, this peptide epitope does not contain serine. Additional research on *Pk*SERA3 Ag2 is needed. 

### 5.4. Uncomplicated Vs. Severe Malaria

If *Plasmodia* are identified, the diagnosis needs to be categorized as uncomplicated or severe malaria. Patients with one or more of the following criteria have severe malaria: “impaired consciousness/coma, severe anemia (hemoglobin < 7 g/dL), acute kidney injury, acute respiratory distress syndrome, circulatory collapse/shock, disseminated intravascular coagulation, acidosis, jaundice (along with at least one other sign of severe malaria)—and/or percent parasitemia of ≥5%” [39]. These patients require IV antimalarial medication [39].

## 6. Rapidly Increasing Numbers of *Pk* Malaria Cases

A 2004 report identified 120 naturally acquired *Pk* malaria cases in Malaysia from 2000 to 2002 [126], which is notable given the last suspected naturally acquired case was in 1971 [127]. In most cases, the parasites were misidentified as *Pm*. Starting in 2018, *Pk* has been the only parasite to cause indigenous malaria in Malaysia [32]. In 2021, Malaysia had 3575 cases of *Pk* malaria, which resulted in 13 deaths [32]. There were also 435 cases in Indonesia, the Philippines, and Thailand [32]. The rise in *Pk* malaria cases has been attributed to deforestation, increased awareness of this parasite, improved diagnostic testing, and a decrease in malaria caused by other species (which reduced immunity to *Plasmodium* species) [23]. There is concern for the human–mosquito–human transmission of *Pk* parasites, which occurs with the other four major human-infecting *Plasmodium* parasites [128]. Thus, *Pk* is considered an emerging threat [23].

## 7. Currently Still No *Pk* Vaccine 

Although AMA-1, *Pk*NBPXa, and *Pk*DBPαII are *Pk* vaccine targets, no *Pk* vaccine is currently available for human use [5,50,57]. In contrast, a *Pf* malaria vaccine known as RTS,S/AS01 (RTS,S or Mosquirix) helps prevent *Pf* malaria in children [129,130]. This vaccine is targeted against the circumsporozoite protein (CSP) on *Pf* sporozoites to prevent liver infection, making it a pre-erythrocytic vaccine. The “RTS” component of RTS,S is a fusion protein. The “R” refers to the central repeat region of CSP, which contains the B cell epitopes. “T” is for the CD4+ and CD8+ T cell epitopes. “S” refers to hepatitis B surface antigen. The second “S” is RTS,S stands for monomeric hepatitis B surface antigens. These “S” monomers assemble with “RTS” into virus-like particles (VLPs) that display the CSP protein [130,131]. AS01 is an adjuvant in the vaccine [132]. 

In 2015, a phase 3 clinical trial of RTS,S in young infants (6–12 weeks old) and children (5–17 months old) was published [132]. The study evaluated whether RTS,S could reduce episodes of clinical *Pf* malaria or severe *Pf* malaria versus a comparator vaccine. Participants received a primary series of three doses of vaccine at 0, 1, and 2 months, followed by a booster at 20 months. In this trial, RTS,S was abbreviated as “R”, and the comparator vaccine was abbreviated as “C”. Participants received either four doses of comparator vaccine (abbreviated “C3C”), a primary schedule of RTS,S followed by a comparator vaccine (“R3C”) or a primary schedule of RTS,S followed by RTS,S (“R3R”). The primary series of comparator vaccine was the meningococcal C conjugate vaccine (Menjugate^TM^) for young infants or the rabies vaccine (VeroRab^TM^) for children. However, the booster of comparator vaccine was Menjuate^TM^ for both young infants and children.

From month 0 to the study end, R3C and R3R vaccine efficacy against episodes of clinical malaria was 28.3% and 36.3% versus C3C, respectively. In young infants, vaccine efficacy was 18.3% and 25.9%, respectively. These vaccine efficacy percentages all had *p* values < 0.0001. From month 0 to the study end, statistically significant vaccine efficacy against episodes of severe malaria was only seen in children receiving R3R, versus C3C, which was 32.2% (*p* value = 0.0009). 

However, there were disappointing outcomes. Vaccine efficacy waned over time. Of the 22 children who developed meningitis during the study, 21 received RTS,S; 11 were in the R3R group, 10 were in the R3C group, and 1 was in the C3C group. Unfortunately, no statistically significant differences in overall mortality, malaria mortality, sepsis, or pneumonia were observed. Critiques of the vaccine have been published [133].

In 2021, the WHO recommended widespread RTS,S vaccine administration in areas with a moderate-to-high transmission of *Pf* [134]. This vaccine is administered as an intramuscular injection into the deltoid. The WHO recommends a 4-dose schedule, with the first dose being administered in children of at least 5 months of age [134,135,136]. The doses should be at least 4 weeks apart, and the fourth dose should be 12–18 months after the third dose [135]. By 2023, at least one vaccine dose was administered to almost 1.7 million children [137]. The vaccine is not thought to provide immunity against *Pk* or the other major human-infecting *Plasmodium* species because it targets a *Pf* protein [138]. *Pk*CSP is being studied and may become a vaccine target [139]. 

## 8. Antimalarial Medications

The treatment of *Pk* malaria can vary due to different guidelines. According to the Ministry of Health in Malaysia, artemether–lumefantrine is first-line treatment for non-severe *Pk* cases [110]. CDC recommends that uncomplicated *Pk* malaria in adult or pediatric patients should be treated with either (1) chloroquine or hydroxychloroquine since there is “no widespread evidence of chloroquine resistance” or (2) artemether–lumefantrine [39,112,140]. 

A study from 2016 found that the half-maximal inhibitory concentration (IC_50_) of chloroquine for *Pk* strain A1-H.1 was 10.9 nM at 24 h and 6.9 nM at 48 h using the [^3^H] hypoxanthine uptake assay [141]. The study authors mentioned that this was similar to the IC_50_ of chloroquine for *Pf* strain 3D7, which was 6 nM at 48 h. Thus, the authors concluded that this *Pk* strain was chloroquine-sensitive. Chloroquine can disrupt *Plasmodium* parasites from degrading hemoglobin to hemozoin, leading to a buildup of heme, which is toxic for *Plasmodium* [62,142,143]. Because *Pk* is chloroquine-susceptible, it may suggest that it needs hemoglobin as a nutrient source. Interestingly, some *Plasmodium* species can replicate in reticulocytes without hemozoin formation, giving them chloroquine resistance [144]. 

Some articles explicitly state that there is no evidence of *Pk* drug resistance [145]. For example, one study compared the in vitro drug susceptibility of *Pk* strain A1-H.1 and *Pf* strain 3D7 for one life cycle [146]. The half maximal effective concentration (EC_50_) was similar for artemether, artesunate, artemisinin, and dihydroartemisinin [146]. The CDC also encourages the hospitalization of *Pk* malaria patients to continuously analyze blood smears every 12–24 h to confirm that antimalarial treatment is causing a decline in parasitemia and to monitor the patient’s clinical status [39,112]. 

In Malaysia, the recommended first-line treatment of severe *Pk* malaria is IV artesunate and oral doxycycline [110]. The CDC recommends treating severe malaria by admitting the patient to the intensive care unit (ICU), calling the CDC, and administering IV artesunate [39,112]. Artesunate is a potent derivative of artemisinin, which is extracted from *Artemisia annua* plants [147]. Tu Youyou received the 2015 Nobel Prize in Physiology or Medicine for her work on artemisinin and malaria. The dose of artesunate is 2.4 mg/kg and needs to be administered at 0, 12, and 24 h. If this medication is not stocked at the healthcare facility, it needs to be emergently requested from a commercial vendor or the hospital’s affiliated distributor [148]. If it cannot be obtained immediately, it can be requested from a nearby hospital, or the patient can be transferred to a hospital that has IV artesunate. Interim treatment needs to be administered, with artemether–lumefantrine being preferred because of the fast onset of artemether (while lumefantrine takes longer to kill the parasites) [39,112,149]. When IV artesunate arrives, it should be started, and the interim treatment should be stopped [148]. 

More than 4 h after the third dose of IV artesunate, parasitemia needs to be measured [39]. (Note: CDC incorrectly used the term “parasite density”, instead of “parasitemia”, in its Malaria Treatment Guidelines [150]). If parasitemia is >1%, IV artesunate must be continued daily for up to six additional days or until parasitemia is ≤1%. If it is ≤1% and the patient can tolerate oral medications, then follow-on oral medications must be administered. If it is ≤1% and the patient cannot tolerate oral medications, then IV artesunate must be continued daily for up to six additional days or until the patient can tolerate oral medications. By using an antiemetic or inserting a nasogastric tube when needed, oral medications might be feasible. Recipients of IV artesunate must be evaluated each week for 4 weeks after starting the mediation for delayed post-artemisinin hemolytic anemia. This assessment can include evaluating the patient’s hemoglobin, haptoglobin, LDH, bilirubin, etc. [39].

## 9. Manual Exchange Transfusion (ET)/Automated RBC Exchange (RBCX)

### 9.1. Manual ET/RBCX

RBCX is an automated procedure in which a trained professional uses an apheresis machine to replace the patient’s reticulocytes and normocytes with healthy donor RBCs. Apheresis literally means “to take away”. Prior to the procedure, the patient’s peripheral or central vasculature needs to be accessed. If there are concerns about the extracorporeal blood volume in the apheresis machine causing hypovolemia (e.g., low-weight or anemic patient), then the apheresis circuitry can be primed with RBC units [151]. Alternatively, anemic patients can be transfused with RBCs prior to starting the procedure. Notably, patient data (i.e., height, weight, hematocrit, etc.) and procedure details (desired post-RBCX hematocrit) can be entered into modern automated, continuous-flow apheresis machines to avoid mistakes and adverse hemodynamic events.

In this procedure, the patient’s blood is anticoagulated and flows through tubing to the apheresis machine, where it can be fractionated into plasma, platelet-rich plasma, leukocytes, and RBCs by a centrifuge. The centrifugal force separates blood according to specific gravity, with RBCs having the highest specific gravity [152,153]. The RBCs (likely including reticulocytes) are selectively removed and donor RBCs are slowly transfused. The procedure usually takes ≤2 h but depends on the multiple factors [154]. For instance, it will take longer if a lower fraction of (host) cells remaining (FCR) is desired (Figure 2C,D).

Manual ET removes the patient’s whole blood and replaces it with donor RBCs reconstituted in albumin solution or plasma. Manual ET can be used if apheresis equipment is not available or there are concerns about the extracorporeal blood volume in the machine representing a high percentage of the patient’s blood volume. Manual ET can be performed using several techniques. For example, a large-bore catheter can be inserted into each vein of the antecubital fossa [155]. One tract serves as the out-flow, in which blood is removed using a blood donation bag. The other tract is the in-flow where RBCs and albumin solution or plasma can be administered. A similar approach can be used, but the out-flow tract can be an artery in the antecubital fossa [156]. The procedure can be isovolumetric [156]. Both manual ET and RBCX can be performed as inpatient or outpatient procedures, and the patient recovery time is typically <1 h.

### 9.2. Manual ET/RBCX for Malaria 

Reports from both developed and developing nations attribute manual ET/RBCX to improved outcomes for malaria patients [155,157,158,159,160,161,162,163,164,165]. For example, one study found that parasite clearance times were significantly shorter in 25 malaria patients who had ET, compared with 31 controls [155]. However, we were unable to find a single report of manual ET/RBCX for *Pk* malaria. In addition, this procedure is rarely used in Africa, possibly because RBCX requires an apheresis machine, which is expensive. The benefits of manual ET may include the removal of (1) proinflammatory cytokines in plasma, such as tumor necrosis factor alpha; (2) iRBCs; (3) parasites; and (4) parasite toxins (e.g., hemozoin and glycosylphosphatidylinositol) [156,166,167]. By removing iRBCs, which can cytoadhere and cause microvascular obstruction, blood viscosity and oxygenation can improve. Manual ET/RBCX for severe malaria is an American Society for Apheresis (ASFA) category III indication—meaning the decision to perform the procedure needs to be individualized [168]. 

Unfortunately, manual ET/RBCX for malaria is rarely carefully reported or evaluated. Descriptions of the specific donor RBCs delivered to malaria patients (i.e., ABO blood type, presence of hemoglobin S, C, E, etc.) are almost always incomplete in case-reports and case-series [156,158,161]. Yet, malaria patients with sickle cell trait, thalassemia trait, blood group O, glucose-6-phosphate dehydrogenase deficiency (G6PDd), and other malaria-resistant RBCs are known to have markedly better clinical outcomes [169,170,171,172,173]. In the 1940s, J.B.S. Haldane proposed the Malaria Hypothesis, in which heterozygotes of hematologic variant alleles (e.g., sickle cell trait) are more likely to survive malaria [174,175,176]. As evidence continues to mount in support of this hypothesis, it is recognized that “As a result of survival advantage against malaria, inherited red cell disorders are the most common monogenic diseases” [177]. 

However, when automated RBCX was used to treat a 13-year-old and two adult patients with CM, sickle cell trait RBC units were deliberately excluded [160]. (Note: Sickle-cell-trait RBC units are typically excluded for patients with hemoglobinopathies, infants, and fetuses requiring intrauterine transfusion [178]). None of the three patients were described as having a history of hemoglobinopathies, so it is unclear why sickle-cell-trait RBCs were excluded. Perhaps the clinicians did not have enough time to test the patients for hemoglobinopathies before the procedure. Delivering malaria-promoting RBCs, because malaria-resistant donor RBCs were unnecessarily excluded, may, unfortunately, substantially reduce the effectiveness of adjunctive manual ET/RBCX for malaria. This same practice was described in multiple cases of RBCX for pediatric patients past infancy without known hemoglobinopathies [159,165]. In one case-series of 3 patients, all tested negative for sickle cell disease [159]. It seems reasonable and prudent to assume—until proven otherwise—that inborn genetic RBC protection against malaria-induced death can be translated into therapeutic protection via RBCX.

Automated RBCX has major advantages over manual ET; it is less labor-intensive, faster, more efficient, and provides better hemodynamic stability [155]. Fortunately, when using a modern automated apheresis machine, the post-exchange hematocrit can be set to the value the clinician feels is the “optimal hematocrit” for the patient—a value that avoids the possibility of malaria-induced hyperviscosity. Manual ET removes whole blood, including plasma and pro-inflammatory cytokines, which may be harmful [179]. A single RBC volume exchange can eliminate about 60% of the patient’s pre-procedure RBCs, and a double exchange can eliminate about 85% [165,168,180]. There are risks with RBC transfusion [181], such as transfusion-transmitted malaria (TTM). However, because the risk of TTM is so low in the US, patients with TTM have been successfully treated with RBCX, which exposes them to additional RBC units [182,183]. Importantly, there is only a slight decline in concentrations of anti-malarial drugs after this procedure [184]. 

## 10. Therapeutically Rational Exchange Transfusion (T-REX) 

T-REX refers to replacing a patient’s iRBCs with special invasion- or disease-resistant donor RBCs instead of nondescript standard-issue RBCs (usually any RBC unit that is ABO and RhD-compatible) that may contain *Pf*-promoting RBCs (such as blood group A, and hemoglobin A RBCs). Of major clinical importance, exposure to RBC alloantigens following pregnancy or transfusion can increase the likelihood of RBC alloimmunization against other alloantigens, which increases the probability of transfusion complications and reduces the availability of compatible RBCs for future transfusion [185,186,187,188,189,190]. As a result, patients who require chronic transfusions often receive RBCs matched for alloantigens beyond ABO and RhD to reduce the likelihood of RBC alloimmunization [191,192,193,194,195]. As patients with sickle cell disease are prone to developing alloantibodies and associated complications following RBC exposure [196,197,198,199,200,201,202], extended phenotype-matching RBC units (i.e., for alloantigens C, E, and K) for RBCX is routinely used when performing these procedures. Given the impact that blood group antigens can have on infectious disease outcomes [203,204,205], the transfusion practices for sickle cell disease provide a precedent for optimizing transfusion for malaria by thoughtfully selecting the most appropriate donor RBCs.

Previously, several T-REX options have been proposed as candidate non-drug adjuncts for *Pf* malaria [206,207,208,209,210,211,212,213] and babesiosis [214]. For example, blood-group-O RBCs have been proposed for use in T-REX to treat *Pf* malaria because blood-group-O patients have better clinical outcomes (including lower mortality), in part due to reduced rosetting (the binding of iRBCs to uRBCs) [170]. It seems prudent to determine if T-REX strategies can decrease morbidity and mortality when (1) appropriate treatment is delayed (e.g., IV artesunate is unavailable), (2) patients are at risk of developing severe malaria, or (3) drug-resistance is suspected. In addition, this non-drug adjunctive treatment may slow the emergence and spread of multidrug-resistant *Plasmodium* strains [215]. 

Here we propose use of *Pk*-resistant RBC variants currently available in blood banks. Blood donors with these RBC variants are eligible to donate RBCs, based on their responses to screening questions, whether they have adequate hemoglobin, testing negative for infectious disease, and other data. Because these variant RBCs are not thought to be harmful, they are not routinely tested for and are part of the blood bank’s inventory.

### 10.1. T-REX of Fy(a-b-) RBCs 

Duffy is a seven transmembrane domain glycoprotein that binds chemokines in the C-X-C and C-C classes (e.g., CXCL10 and CCL2) [216,217]. The letters “C” and “X” refer to N-terminal cysteines or “other” amino acids, respectively. The FY*A allele leads to Fy^a^ expression, while the FY*B allele, caused by c.125G>A (coding DNA mutation at nucleotide 125 from guanine to adenine), leads to the expression of Fy^b^. Fy(a-b-) is the most common phenotype in Blacks and is usually caused by the mutation c.-67T>C in the erythroid promoter GATA-1-binding motif of FY*B. This allele is known as FY*B^ES^, in which “ES” stands for “erythrocyte silent” [216]. This mutation blocks Fy^b^ expression on RBCs only, but not other tissues. In contrast, Fy(a-b-) phenotype is extremely rare among White and Asian populations. 

*Pk* parasites need to bind to the Duffy antigen [5,218,219] on the RBC surface before invading RBCs [61,220,221]. Therefore, *Pk* invasion-resistant Duffy-negative RBCs, also known as Fy(a-b-), could be delivered via manual ET/RBCX to prevent or treat severe malaria. However, three studies found that 0% of Malaysians had the Fy(a-b-) phenotype [218,222,223]. In addition, there was no statistically significant difference in Fy^a^ and Fy^b^ expression in *Pk* malaria patients versus controls [218,223]. The Fy(a-b-) phenotype is found in less than 5% of individuals in SEA or Asia [224,225]. Thus Fy(a-b-) RBC units would be difficult to obtain in this region. 

It is important to consider that Malaysia has approximately 25 million visitors annually [12]. A 2014 study reviewed more than 66 articles of travelers acquiring *Pk* malaria [12]. So, clinicians across the world should be aware that febrile patients who traveled to Malaysia or neighboring countries may have *Pk* infection [226]. Interestingly, “The first natural infection of *P knowlesi* in a human was reported in 1965 in a man who returned to the USA after visiting peninsular Malaysia” [126]. The Fy(a-b-) phenotype is most common in Africa. One study found this phenotype in 6091 (53.6%) of 11,370 persons [225]. While no *Pk* cases have been reported in Africa, there is concern about *Pk* malaria patients presenting for care on the continent and the potential for misdiagnosis [227,228]. 

The Fy(a-b-) phenotype can be prevalent outside of Africa. Studies have found the Fy(a-b-) phenotype in 78.32% of southwestern Saudi Arabian blood donors [229]; 61% of eastern Saudi Arabian blood donors [230]; 48.69% of blood donors in south Gujarat, India [231]; and 48% of “black blood-donors in Southwestern Colombia” [232]. In addition, *Pk* malaria has been documented in four Indian states (Uttar Pradesh, Delhi, Bihar, Andaman, and Nicobar) [233,234,235].

One study found that 82.76% of the Jarawas tribe of the Andaman and Nicobar Islands were Fy(a-b-) [236]. Another study found that 11.9% of malaria patients on these islands were infected with *Pk* [235]. In addition, *Pk* was also found in *Anopheles* mosquitoes from these islands, which also harbor *M. fascicularis* [237]. By phenotyping RBCs from the RBC unit tubing segments using standard anti-Fy^a^ and anti-Fy^b^ blood typing antibodies, Fy(a-b-) RBC units could be identified and used for anti-*Pk* T-REX. 

### 10.2. T-REX of G6PDd RBCs 

G6PD is an enzyme in the pentose phosphate pathway (PPP), which leads to reduction of nicotinamide adenine dinucleotide phosphate (NADP+) to NADPH [59,238]. This keeps glutathione in a reduced state to protect against oxidative damage. RBCs can only synthesize NADPH via the PPP, so G6PDd makes RBCs more sensitive to oxidative stress. G6PDd is an X-linked condition and considered to be one of the most common human enzyme deficiencies [59]. 

If Fy(a-b-) RBC units are scarce in given region (e.g., SEA), healthcare providers may consider the anti-*Pk* T-REX of G6PDd RBCs because they may reduce the risk of severe *Pk* malaria. A case–control study examined *Pk* malaria patients that presented to two primary referral hospitals in Malaysia from 2012 to 2015 [82]. The study included 229 patients with symptomatic *Pk* malaria mono-infections (confirmed by PCR) and 683 controls. G6PDd was present in 2 (0.9%) of 227 *Pk* malaria patients who were tested and 43 (6.5%) of 663 controls that were evaluated. The adjusted odds ratio was 0.20, and the *p* value was 0.045. To further support its potential benefit, there is evidence that the G6PDd phenotype was selected by human evolution because it reduces the risk of severe malaria and death [239,240,241]. 

Additional studies showing improved outcomes of G6PDd individuals with *Pk* malaria would strengthen the case for the T-REX of G6PDd RBCs. One study looked at baseline features of malaria patients [65]. G6PDd was found in 2.6%, 4.3%, and 5.6% of children < 12 years of age infected with *Pk*, *Pf*, *and Pv*, respectively. In addition, G6PDd was found in 1.1%, 4.3%, and 2.1% of adults infected with *Pk*, *Pf*, *and Pv*, respectively. Interestingly, G6PDd was least common in *Pk* patients of both age groups. It is worth noting that primaquine can trigger hemolysis in G6PDd individuals. However, this medication is not typically used to treat *Pk* malaria [39,112,242,243]. However, some *Pk* malaria patients (e.g., those co-infected with *Pv* and/or *P. ovale*) may be treated with primaquine to prevent relapse due to hypnozoites in the liver [233,243]. 

The WHO recently reclassified G6PD variants into four classes. Persons in Class A, B, C, and U have median G6PD activity <20%, <45%, 60–150%, and 45–60%, respectively [244]. Class C has no hemolysis, while Class B is associated with acute hemolysis, when there is a trigger (e.g., infection, consuming fava beans, or taking certain medicines). Thus, many G6PDd persons will go their entire life without symptoms or complications and never know they were G6PDd [244]. However, Class A is associated with chronic non-spherocytic hemolytic anemia. There are multiple factors that determine G6PD activity, including the exact genetic variant (>230 known) and whether the individual is male or female (X-linked condition). 

Because most G6PDd individuals do not have chronic anemia, they have a hemoglobin level that is sufficient to allow for blood donation. Thus, persons with G6PDd donate blood in many countries. For example, about 7.6% of Thai blood donors, more than 16% of Iranian blood donors, and 25.5% of Nigeria blood donors were G6PDd [245,246,247,248]. In SEA and the surrounding area, G6PDd can be found in about 9% in the Negrito tribe of the Malaysian Orang Asli, 10.5% in the Chiang Mai Province of Thailand, 16.6% in the South Central Timor district of Eastern Indonesia, 25.0% in one region of Myanmar, as high as 25.7% in the Philippines, and 29.6% of the Kachin ethnic group at the China–Myanmar border [249,250,251,252,253,254]. In Malaysia and Thailand, the point-of-care fluorescent spot test (FST) has been used to detect G6PDd blood donors [248,255]. Because RBCs in the blood bag have G6PD activity, which correlates with the attached tubing segments [256], RBCs from the tubing segments can be tested using the FST to identify G6PDd RBC units for anti-*Pk* malaria T-REX.

The FST test starts by incubating blood with NADP and glucose-6-phosphate [257]. G6PD in the blood will lead to the production of 6-phosphogluconate and NADPH (which is fluorescent). This sample is then spotted onto filter paper. After it dries, it is viewed under UV light. The fluorescent intensity is proportional to the G6PD activity. 

The safety of transfusing G6PDd RBCs needs to be considered. The WHO states that G6PDd blood donors should be allowed to donate if do not have a history of hemolysis [258]. However, they add that G6PDd RBCs should not be used for intrauterine transfusion of fetuses, neonatal ET, or transfusion of G6PDd individuals [258]. This is because G6PDd RBC units have been shown to have a reduced post-transfusion recovery. The 24 h post-transfusion recovery of the autologous transfusion of G6PDd versus non-G6PDd RBCs stored in additive solution formula 3 (AS-3) was 81.0% versus 86.8%, respectively [259]. The authors of the study state that the clinical consequences of this decline are unknown. They also mention the Food and Drug Administration (FDA) requirement that 75% of the RBCs must survive 24 h after being transfused [260]. Despite the decreased survival of transfused G6PDd RBCs, the FDA requirements were fulfilled. 

The safety of transfused G6PDd RBCs has been studied in SEA. In 2022, there was a randomized controlled clinical trial of G6PDd versus non-G6PDd RBC units transfused into patients with hypoproliferative anemia in Thailand [261]. The transfusion of G6PDd RBC units was associated with “mildly elevated indirect bilirubin after transfusion” [261,262] and decreased survival. However, there were no statistically significant differences in the change of hemoglobin, hematocrit, LDH, and haptoglobin between the groups, and long-term survival differences may not be clinically important when treating an acute infection [261]. In addition, no clinical symptoms or transfusion reactions were noted [261]. This data should alleviate some concerns about transfusing G6PDd RBCs. Furthermore, an African study showed that the manual ET of G6PDd RBCs (RBCs prevalent in malaria-endemic Nigeria) was safe and effective in hospitalized neonates, as there was no statistically significant difference in the post-procedure hemoglobin, hematocrit, serum bilirubin, or reticulocyte count [263]. Still, the risks and benefits of transfusing G6PDd RBCs in malaria patients should be carefully considered. 

### 10.3. T-REX of Southeast Asian Ovalocytes

Southeast Asian Ovalocytosis (SAO) is caused by a 9 amino acid deletion in the band 3 protein (AE1), which is an anion exchange protein on RBCs [264,265]. Band 3 transports one Cl^-^ anion in the opposite direction of an HCO_3_^−^ ion across the RBC membrane [266]. Heterozygous SAO is mostly asymptomatic, but there is less efficient gas exchange, while homozygous SAO is a severe disease (with severe anemia) [267]. Thus, only heterozygous SAO is considered here. SAO RBCs do not have normal biconcave disc morphology (i.e., anucleate cells with a round area of central pallor). They can be macro-ovalocytes (large oval shapes), stomatocytes (slit-like area of central pallor), or stomato-ovalocytes [268]. SAO was thought to have evolved due to it offering protection against *Pv* and CM caused by *Pf* [210,264,269].

One in vitro study showed that the *Pk* invasion of RBCs from persons with SAO, also known as Melanesian elliptocytosis [270], was “markedly reduced” compared to controls [271]. In the first experiment, there were 20.6, 14.2, and 17.9 *Pk* ring forms per 100 RBCs for the controls and 0.4 and 0.5 for two SAO samples. A similar trend was noted in the second experiment. Importantly, all SAO RBCs and control RBCs expressed the Duffy antigen. The study also showed that SAO RBCs had fewer *Pk* merozoites attached to them, compared to the controls. However, only five samples were tested, and the findings have not been confirmed by others. 

The prevalence of SAO can be substantial in parts of Malaysia, the Philippines, Indonesia, Thailand, and Papua New Guinea [269]. The vast majority of individuals with SAO do not have hemolytic anemia, so they are likely to be eligible for blood donation. However, the prevalence of SAO RBCs in blood banks is not well-studied. SAO RBC units could be identified using blood bag tubing segments to create a blood smear to identify the unique RBC appearance [210]. To our knowledge, blood donors are not routinely tested for SAO, and persons with SAO are not deferred from donating. Clinical studies showing improved outcomes of individuals with SAO with *Pk* malaria would strengthen the case for T-REX of SAO RBCs. Table 1 shows a summary of RBC variants that might be *Pk*-resistant.

### 10.4. Performing Anti-Pk T-REX

To perform anti-*Pk* T-REX, healthcare professionals will need to ask blood bank staff if they can provide Fy(a-b-), G6PDd, and/or SAO RBC units. Using tubing segments from RBC units, Fy(a-b-), G6PDd, and SAO RBCs can be identified using standard blood-typing reagents, the rapid FST test, and based on their unique microscopic appearance, respectively. The availability of these units depends on the blood donor population demographics and the geographic location of the blood bank. Importantly, the identification of these *Pk*-resistant RBC units can be concomitant as the patient awaits and then obtains the vascular access needed for the procedure. 

The RBC units should be transfused in a specific order. Manual ET/RBCX slowly and continuously removes RBCs from the patient, while RBC units are slowly and continuously transfused. So, the first transfused RBC unit will have a higher percentage of RBCs removed than the last unit transfused. If only a limited number of *Pk*-resistant RBC units can be obtained, it is advisable to start with transfusion of standard-issue RBCs at the beginning of the procedure, followed by *Pk*-resistant RBCs at the end. This sequence will minimize the removal of the *Pk*-resistant RBCs during the procedure. 

### 10.5. Timeline of Therapeutic Benefits of Anti-Pk T-REX

It is important to consider the timing of *Pk*’s asexual replication, the therapeutic benefit of T-REX and antimalarials, and patient deaths. If T-REX of Fy(a-b-) RBCs is performed, then the circulating uRBCs and iRBCs will be removed during the ~2 h procedure. Because sequestered iRBCs adhere to the endothelium, they will not be removed by T-REX. However, over the next 24 h (one asexual life cycle), the schizonts will rupture, releasing merozoites and reducing sequestration. The merozoites will not be able to invade Fy(a-b-) RBCs. Thus, the benefits of T-REX would be realized during the procedure, within 24 h (one asexual lifecycle), and during subsequent asexual life cycles. This is important because a case-series about *Pk* malaria fatalities found that the median time from admission to death was 41 h [41]. So, T-REX would likely provide a therapeutic benefit before it is too late. 

The timing of the benefit of antimalarials is complex. This topic came up in the South East Asian Quinine Artesunate Malaria Trial (SEQUAMAT) and African Quinine Artesunate Malaria Trial (AQUAMAT), in which clinical outcomes from quinine and artesunate treatment were compared in patients with severe *Pf* malaria [272,273]. Both quinine and artesunate can target sequestered iRBCs, but only artesunate can kill ring stages in circulating iRBCs, which prevents them from maturing and sequestering in venules [272,273]. This may explain why clinical outcomes were better with IV artesunate than IV quinine. The effectiveness of an antimalarial is thought to depend on how well the drug can kill parasites, if killed parasites in iRBCs can still remain in the blood vessels, and if killed parasites in iRBCs can still cytoadhere or sequester [274]. A study discovered that despite *Pf* parasites inside of RBCs being killed by drugs, these iRBCs can still cytoadhere for hours because *Pf*EMP-1, a key molecule this process, remains on the surface of iRBCs [275]. So, parasite clearance (i.e., iRBC clearance) may be a better measure of benefit than parasite death. In addition, drug resistance, which varies based on location, can slow the rate of parasite clearance [276]. A randomized controlled clinical trial of parasite clearance in Ugandan children treated with IV artesunate found that the median time to clear half of the parasites was 4.8 h, suggesting that artemisinin resistance had not developed in this region [277]. Still, RBCX, which typically takes ~2 h, can remove more than half of the patient’s RBCs. Thus, the kinetics of RBCX seem faster than IV artesunate. 

### 10.6. After Anti-Pk T-REX

After the patient’s malaria resolves, there is no need to perform RBCX to remove the transfused Fy(a-b-), G6PDd, or SAO RBCs. This is because these RBCs were donated by healthy non-anemic persons who met blood donor eligibility requirements. However, the duration of survival of Fy(a-b-), G6PDd, and SAO RBCs is worth discussing. Healthy human RBCs can circulate for about 120 days [278]. Fy(a-b-) RBCs should be able to circulate for a long length of time because this phenotype is not associated with hemolysis. However, G6PDd and SAO RBCs may have reduced survival compared to Fy(a-b-) RBCs, but their exact lifespan appears to be unknown [278,279]. 

If healthcare professionals publish anti-*Pk* T-REX case reports with RBCX parameters and donor and recipient RBC variables, its effectiveness could be evaluated in a meta-analysis. For example, a 75 kg O+ non-G6PDd patient with *Pk* malaria may receive automated T-REX using two standard-issue O+ RBC units, followed by two O+ G6PDd RBC units, and then two O+ Fy(a-b-) RBC units. The patient’s clinical status and laboratory values (e.g., hematocrit, parasitemia) before and after T-REX can be reported. For a more complete list of procedure parameters to report, view reference [280].

## 11. ABO Blood Group and *Pk* Malaria

Blood group O has been shown to reduce the risk of severe *Pf* malaria, which likely explains its high prevalence in *Pf* malaria “hot-zones” [173,209,281]. We were only able to identify one study that collected data on blood-group-O *Pk* malaria patients [66]. This study found blood group O in 28%, 12.5%, and 9.5% of *Pk*, *Pf*, *and Pv* malaria patients admitted to a Malaysian hospital. However, the prevalence of blood group O in non-malaria patients was not measured. So, it is not known if blood group O provides some benefit to *Pk* malaria patients. Studies assessing the impact of ABO blood group on *Pk* malaria would be beneficial. If blood group O protects against severe *Pk* malaria, it could be a feasible anti-*Pk* malaria T-REX option. This is because (1) blood group O is a “universal” blood type, (2) it is found in about 30% or more of the global population, and (3) no additional testing is required. 

## 12. Conclusions

Anti-*Pk* T-REX is a theoretical optimization of manual ET/RBCX, in which *Pk*-iRBCs are replaced with *Pk*-resistant RBCs. Fy(a-b-) and SAO RBCs are likely to resist *Pk* invasion, while G6PDd RBCs are likely to reduce the risk of severe *Pk* malaria. Anti-*Pk* T-REX may be beneficial if IV artesunate or other antimalarial medications are not readily available, if patients are likely to progress to severe disease, or if parasite drug-resistance develops. 

## Figures and Tables

**Table 1:** RBC variants which are likely to provide resistance against *Pk* malaria.

RBCs	Evidence for Resistance against *Pk*
Fy(a-b-)	Duffy is an essential receptor for *Pk* invasion. No *Pk* malaria patient has the Fy(a-b-) phenotype. Invasion assays show a decline in *Pk* invasion of Fy(a-b-) RBCs. Offers a survival advantage against *Pv* malaria.
G6PDd	G6PDd individuals are less likely to present to the hospital with *Pk* malaria because they are more likely be symptomatic. Likely provides a survival advantage against malaria.
SAO	An invasion assay showed a marked reduction in *Pk* parasite invasion. Probably offers a survival advantage against *Pf* malaria.

## Abbreviation

AKI = Acute kidney injury, AMA 1 = apical membrane antigen 1, ARDS = acute respiratory distress syndrome, ASFA = American Society for Apheresis, CDC = Centers for Disease Control and Prevention, CM = cerebral malaria, CSP = circumsporozoite protein, DBP = Duffy-binding protein, ET = exchange transfusion, FCR = fraction of cells remaining, FST = fluorescent spot test, Fy = Duffy, Fy(a-b-) = Duffy-negative, G6PDd = glucose-6-phosphate dehydrogenase deficiency, h = hour(s), iRBC = infected red blood cell, IV = intravenous, LDH = lactate dehydrogenase, NBP = normocyte-binding protein, PCR = polymerase chain reaction, *Pf = Plasmodium falciparum*, *Pk = Plasmodium knowlesi*, *Pm = Plasmodium malariae*, *Pv = Plasmodium vivax*, RBC = red blood cell, RBCX = red blood cell exchange, RDT = rapid diagnostic test, RTS,S = *Plasmodium falciparum* malaria vaccine, SAO = Southeast Asian ovalocytosis, SEA = Southeast Asia, SICA = schizont-infected cell agglutination, T-REX = therapeutically rational exchange, uRBC = uninfected red blood cell, WHO = World Health Organization.
